# Supplementary material for: Kinesin-8-specific loop-2 controls the dual activities of the motor domain according to tubulin protofilament shape
Source: Nat Commun. 2022 Jul 20;13:4198. doi: 10.1038/s41467-022-31794-3 (PMC9300613; doi:10.1038/s41467-022-31794-3)
Supplement: Supplementary file 1 — Supplementary Information [file 41467_2022_31794_MOESM1_ESM.pdf]

Kinesin-8-specific loop-2 controls the dual activities of the motor domain according to tubulin protofilament shape

Byron Hunter[1*], Matthieu P.M.H. Benoit[2*], Ana B. Asenjo[2], Caitlin Doubleday[1], Daria Trofimova[1], Corey Frazer[3], Irsa Shoukat[1], Hernando Sosa[2†] and John S. Allingham[1†]

[1]Dept. of Biomedical and Molecular Sciences, Queen's University, Kingston, ON K7L 3N6, Canada
[2]Dept. of Biochemistry, Albert Einstein College of Medicine, Bronx, NY 10461, USA
[3]Department of Molecular Microbiology and Immunology, Brown University, Providence, Rhode Island, 02912, USA
*These authors contributed equally to this work
[†] Corresponding authors. Email: hernando.sosa@einsteinmed.edu and allinghj@queensu.ca

Supplementary Information

Supplementary Table 1. Names, genotypes, mating types, and sources of the strains used in this study

Strain	Genotype (brief)	Genotype (full)	Mating type	Parent
CF027	Wild type	*his1*$^{-/-}$ *leu2*$^{-/-}$ *arg4*$^{-/-}$	α/α	RBY1132[1]
CF239	*kip3* +/Δ	*kip3::LEU2*$^{+}$*/KIP3*$^{+}$ *his1*$^{-/-}$ *leu2*$^{-/-}$ *arg4*$^{-/-}$	α/α	CF027
CF254	*kip3* Δ/Δ	*kip3::LEU2*$^{+}$*/ kip3::HIS1*$^{+}$ *his1*$^{-/-}$ *leu2*$^{-/-}$ *arg4*$^{-/-}$	α/α	CF239
CF360	*kip3 Δ/Δ+KIP3*$^{+}$	*kip3::LEU2*$^{+}$*/ kip3::HIS1*$^{+}$*::pCIp10-KIP3-ARG4*$^{+}$ *his1*$^{-/-}$ *leu2*$^{-/-}$ *arg4*$^{-/-}$	α/α	CF254
CF370	Tub2-mCherry	*NEUT5L::[pGAL1-TUB2-mCherry-ARG4]/NEUT5L*$^{+}$<br>*his1*$^{-/-}$ *leu2*$^{-/-}$ *arg4*$^{-/-}$	α/α	RBY1132[1]
CF363	Tub2-mCherry<br>SPC98-GFP	*NEUT5L::[pGAL1-TUB2-mCherry-ARG4]/NEUT5L*$^{+}$<br>*SPC98*$^{+}$*/SPC98-GFP-SAT1*<br>*his1*$^{-/-}$ *leu2*$^{-/-}$ *arg4*$^{-/-}$	α/α	CF370[2]
CF376	Tub2-mCherry<br>*kip3* Δ/Δ	*NEUT5L::[pGAL1-TUB2-mCherry-ARG4]/NEUT5L*$^{+}$<br>*kip3::LEU2*$^{+}$*/ kip3::HIS1*$^{+}$ *his1*$^{-/-}$ *leu2*$^{-/-}$ *arg4*$^{-/-}$	α/α	CF254
CF379	Tub2-mCherry<br>SPC98-GFP<br>*kip3* Δ/Δ	*TUB2-mCherry-ARG4*$^{+}$*/ TUB2*$^{+}$ *SPC98-GFP-SAT1/SPC98*$^{+}$<br>*kip3::LEU2*$^{+}$*/ kip3::HIS1*$^{+}$ *his1*$^{-/-}$ *leu2*$^{-/-}$ *arg4*$^{-/-}$	α/α	CF376
CF171	Tub2-GFP	*NEUT5L::[pGAL1-TUB2-GFP-SAT1]/NEUT5L*$^{+}$ *his1*$^{-/-}$ *leu2*$^{-/-}$ *arg4*$^{-/-}$	α/α	CF026[2]
CF217	Tub2-GFP<br>*kip3* +/Δ	*NEUT5L::[pGAL1-TUB2-GFP-SAT1]/NEUT5L*$^{+}$<br>*kip3::LEU2*$^{+}$*/KIP3*$^{+}$ *his1*$^{-/-}$ *leu2*$^{-/-}$ *arg4*$^{-/-}$	α/α	CF171
CF230	Tub2-GFP<br>*kip3* Δ/Δ	*NEUT5L::[pGAL1-TUB2-GFP-SAT1]/NEUT5L*$^{+}$<br>*kip3::LEU2*$^{+}$*/ kip3::HIS1*$^{+}$ *his1*$^{-/-}$ *leu2*$^{-/-}$ *arg4*$^{-/-}$	α/α	CF230

Supplementary Table 2. Oligonucleotide primers used in strain construction

Primer	Purpose	Sequence (5' to 3')
P122	Long homologous tail knock-out primer 5' *KIP3::HIS1⁺/LEU2⁺*	AATCCTTCTCATAAAAATCAATTAACAAATTCATT-GTGAAACAACTTAGCCGCATTTGAT-TCCTATGTTACCAGTGTGATGGATATCTGC
P123	Long homologous tail knock-out primer 3' *KIP3::HIS1⁺/LEU2⁺*	TTTCCTATTATAAATCGATCAACTATAATATAAGAAA-GCTTAACTTTGACTTTTTGACTTT-GATTTCGAAGCTCGGATCCACTAGTAACG
P11	*HIS1⁺* check right forward	AACACAACTGCACAATCTGGC
P12	*HIS1⁺* check left reverse	ATTAGATACGTTGGTGGTTCAGTT
P13	*LEU2⁺* check left reverse	AGAATTCCCAACTTTGTCTGTTC
P16	*ARG4⁺* check left reverse	TTCCATTTAGAGAAACTCATCATATTT
P17	*SAT1⁺* check left reverse	CATACCACCGTCCATTTTGAATG
P18	*SAT1⁺* check right forward	TGATGAAGACTCTGCTTGCTATG
P124	-500 bp *KIP3* check	CAAGCAGGATGATAATTATCAATCAATCAGTCGG
P130	*KIP3* ORF +/- 1000 bp into pCIP10 forward (SalI)	GGACCGGTCGACAGGTGCTGAAGATGTTCGAAGTGATGAA
P131	*KIP3* ORF +/- 1000 bp into pCIP10 reverse (KpnI)	GGACCGGGTACCCACTTATCTCCTCTCTTCCTCTCTTTCTCTCTC
P107	*pGAL1-Tub2-GFP/mCherry* downstream check 3'	TATTATCTATATTGTCAAGCCAAGACAAGCCCATT
P108	*SPC98-GFP:Sat1* Forward longtailed primer	TTTGAAAAATGATTTGAATAGAGATTATAATTTAAAGGATCTTAG-TAAGTTGTTAGGTGGTGGTTCTAAAGGTGAAGAATTATTCACTGG
P109	*SPC98-GFP:Sat1* Reverse longtailed primer	TGAGCTTTACAGAGATCTTGTCGGTAATCATAGATTTCCCCACTT-GTTCTGTAATCGACGAAATTGAGGACCACCTTTGATTGTAAATAG
P110	*SPC98-GFP* integration check right reverse	GCAGCGTCCACCCTTTGTAAAAGTG

Supplementary Table 3. Extra data collection and refinement parameters

Dataset	Microscope used	Pixel Size (Å)	Box size of symmetric map (pixels) [a]	Magnification anisotropy % [b]	Magnification anisotropy angle [b]	Box size (pixels) [c]
MT-*Ca*Kip3-MDC-ANP	Krios 3	0.825	664	0.54	48.4	416
MT-*Ca*Kip3-MDC-AAF	Krios 2	0.849	664	1.50	178.3	416
MT-*Ca*Kip3-MDC-APO	Krios 3	0.825	664	0.71	46.7	416
MT-*Ca*Kip3-MDN-ANP	Krios 1	0.830	664	0.46	83.7	416
MT-*Ca*Kip3-$\mathrm{MDN}_{\mathrm{L2}\text{-}\mathit{Hs}\mathrm{KHC}}$-ANP	Krios 2	1.090	512	1.16	177.2	320
MT-*Ca*Kip3-$\mathrm{MDN}_{\mathrm{L2}\text{-}\mathit{Hs}\mathrm{KHC}}$-APO	Krios 2	0.849	664	1.50	177.7	416
CT-*Ca*Kip3-MDC-ANP	Krios 3	1.083	700	n.c.	n.c.	416

[a] Box size used during the helical refinement of the microtubule datasets (MT) or during the full C14 ring refinement (CT-*Ca*Kip3-MDC-ANP). The following local refinements use a smaller box size (see Note *c*).
[b] Given when estimated and corrected for a given dataset. n.c.: magnification anisotropy not corrected.
[c] Box size used for the local refinements and final reconstructions.

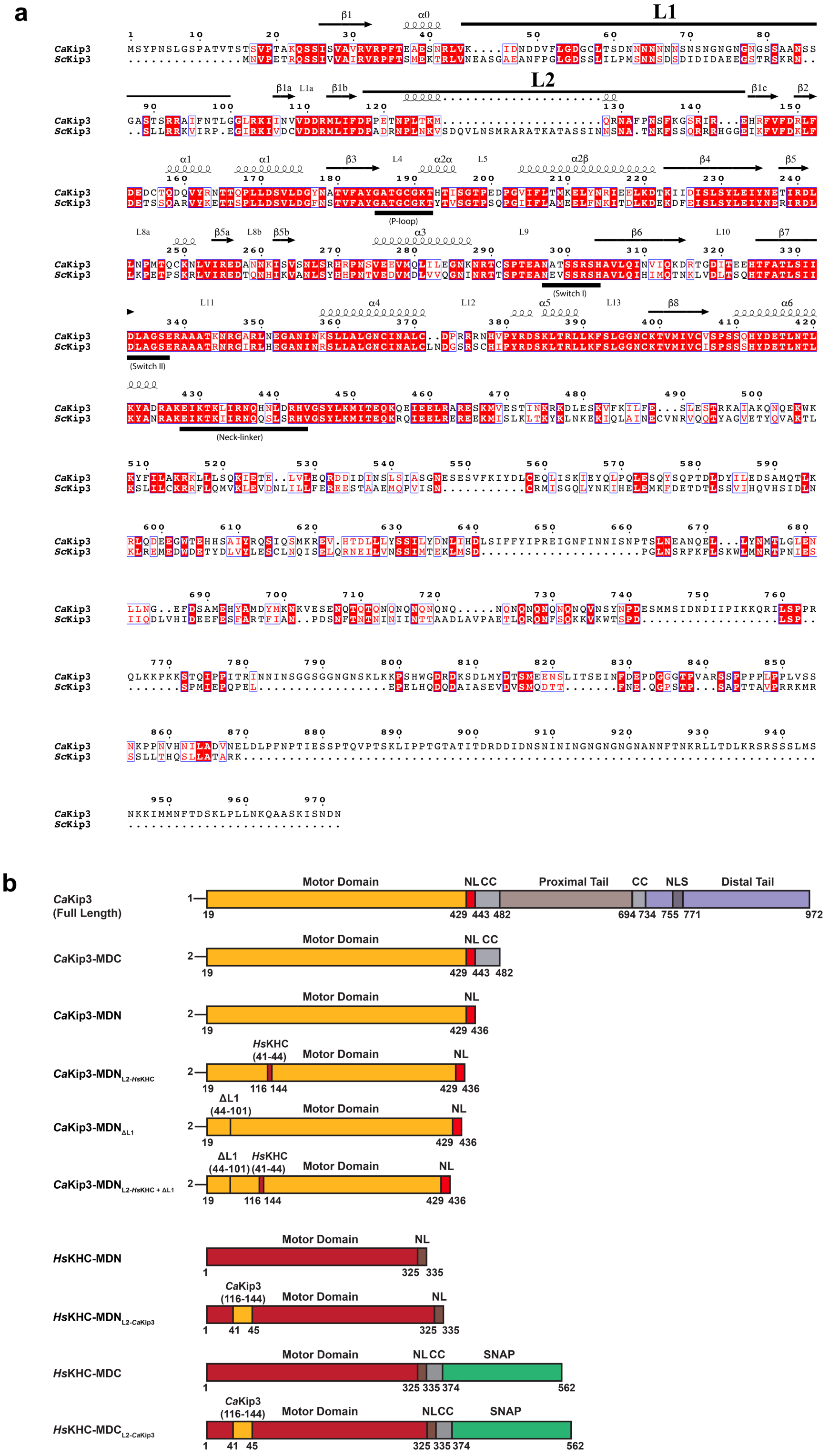

Supplementary Fig. 1: Alignment of *Ca*Kip3 and *Sc*Kip3 protein sequences and schematic of *Ca*Kip3 and *Hs*KHC constructs. (a) The numbering relates to residues of *Ca*Kip3. Identical residues are colored in white on a red background; similar residues are colored in red font. The sequence alignment was performed using Needle at the EMBL-EBI[3]. Secondary-structure elements are placed above the sequence alignment according to their positions in the crystal structures of *Ca*Kip3 (PDB entry 7LFF) using ESPript[4]. The loop-1, loop-2, the ATP-binding pocket, and the switch regions are indicated. (b) Schematic illustration of the structural motifs in *Ca*Kip3 and the truncation sites for all *Ca*Kip3 and *Hs*KHC constructs used in this study. The neck-linker (NL), predicted coiled-coil forming regions (CC), predicted nuclear localization sequence (NLS), predicted proximal and distal regions of the *Ca*Kip3 tail domain, and SNAP tag sequence are shown.

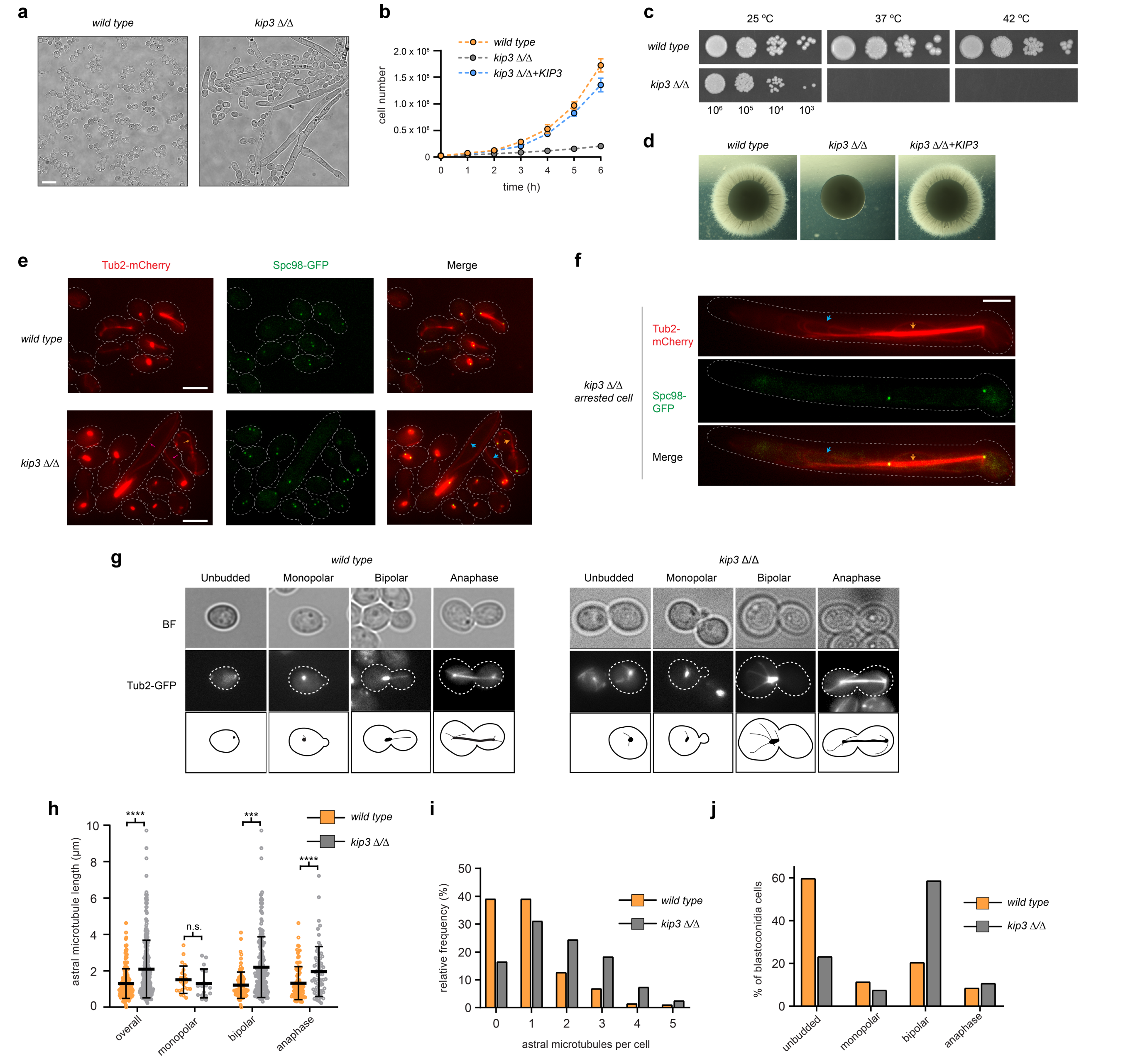

Supplementary Fig. 2: *Ca*Kip3 loss causes growth defects and longer microtubules in *Candida albicans*. (a) Representative images of logarithmically growing *wild type* and *kip3* Δ/Δ cells. Similar results were obtained from three independent experiments. Scale bar = 10 µm. **(b)** Growth rates of cells diluted to 2.5 x 10^6 cells/mL and grown in SDC medium at 30 °C. The number of cells/mL was counted every hour with a hemocytometer. Data presented as mean ± SD (n=3 independent experiments). **(c)** Temperature-dependent growth of 5 µL drops of cells diluted as indicated, and then plated on YPD and incubated for 48 hours. **(d)** Images of cells plated on Spider medium and incubated at 30 °C for 5 days. **(e)** Representative images of *wild type* and *kip3* Δ/Δ cells expressing tubulin (Tub2) labelled with mCherry and spindle pole body protein Spc98 labelled with GFP. Cells were grown in SDC media at 25 °C. Long astral microtubules (blue arrows) and hyper-elongated “fishhook” spindles (orange arrow) are indicated. Dashed lines outline individual cell boundaries. Similar results were obtained from three independent experiments Scale bar = 5 µm. **(f)** Representative images of an arrested *kip3* Δ/Δ cell showing a hyper-elongated spindle (orange arrow) and long, looping astral microtubules (blue arrow). Scale bar = 5 µm. **(g)** Representative images of *wild type* and *kip3* **Δ/Δ** cells expressing Tub2-GFP are shown beside cartoon illustrations of each cell to illustrate how cells were grouped according to their cell and spindle morphologies. **(h)** Astral microtubule lengths from logarithmically growing wild type (n = 199 microtubules from 126 cells) and kip3 Δ/Δ cells (n = 289 microtubules from 137 cells) expressing Tub2-GFP were examined over 3 independent experiments. Measurements were performed on blastoconidia and involved tracing the path of the astral microtubule from the spindle pole to the plus end. Cells exhibiting polarized growth were excluded. Center line and limits represent mean and SD, respectively. Asterisks indicate two-tailed unpaired t-test significance: p=1.4e-10 (overall), p=0.4 (monopolar), p=3.0e-7 (bipolar) and p=6.0e-4 (anaphase). **(i)** Frequency distribution for the number of astral microtubules per cell for *wild type* (n = 205 cells) and

kip3 Δ/Δ (n = 168 cells). **(j)** Quantification cell and spindle morphology of *wild type* (n = 509 cells) and *kip3* Δ/Δ (n = 215 cells) strains expressing Tub2-GFP, as depicted in **(g)**. Source data are provided as a Source Data file.

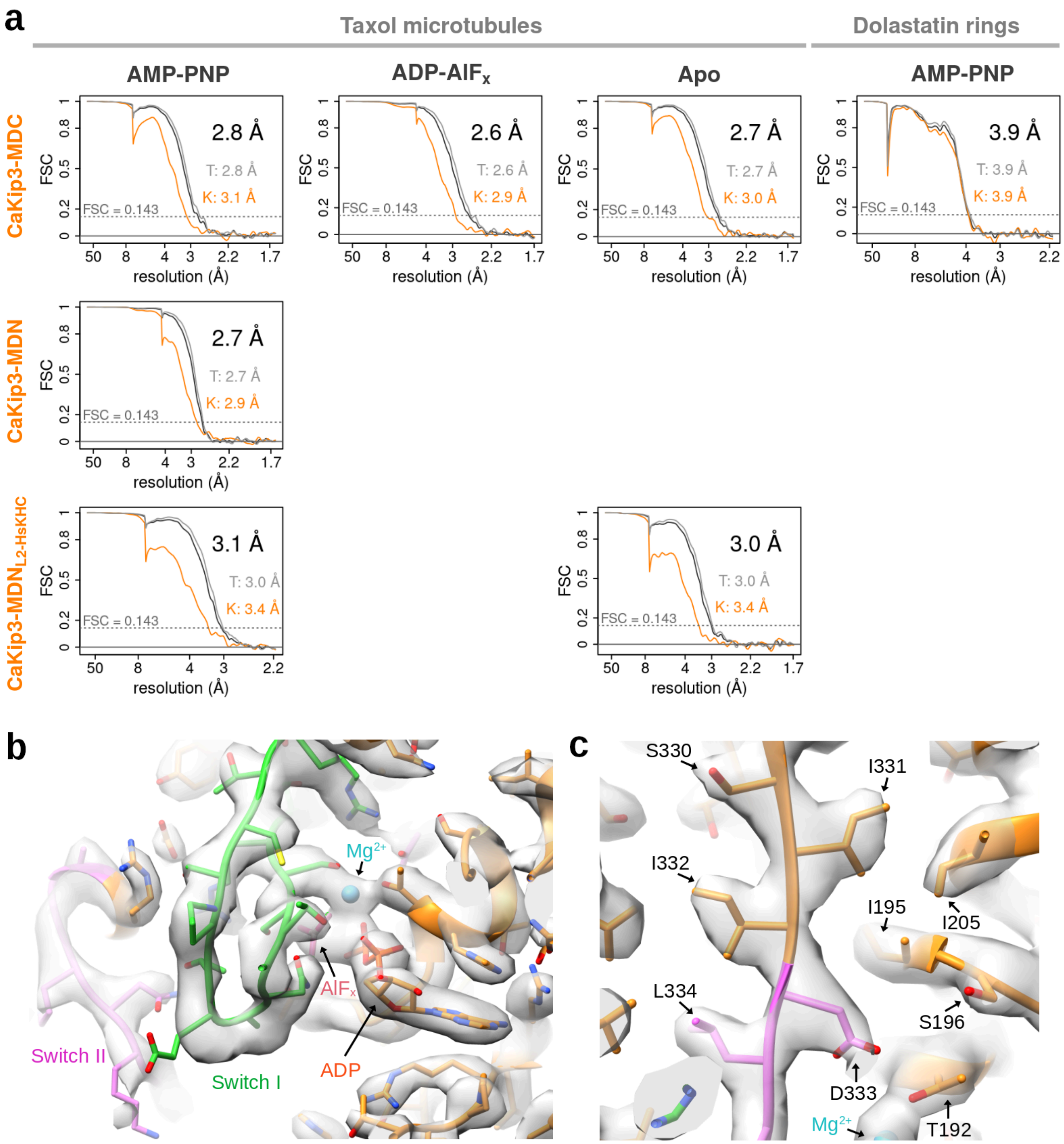

Supplementary Fig. 3: Resolution estimation of the cryo-EM maps. (a) FSC curves for each of the structures solved by cryo-EM. Overall FSC in black, tubulin part FSC in grey and kinesin part FSC in orange. Resolution values ($FSC_{0.143}$) for the overall, tubulin (T) and kinesin (K) parts are indicated. Half maps and masks used to generate the FSC curves are deposited in the EMDB (accession numbers in Table 2). **(b)** Iso-density surface representation of the MT-*Ca*Kip3-MDC-AAF map with underlying model, showing the well resolved nucleotide pocket area of the kinesin in the closed state. The key elements, Switch I, Switch II, ADP, Mg^{2+} and AlF_x, are labeled. **(c)** Inset of the MT-*Ca*Kip3-MDC-AAF map showing a strand of the central beta-sheet of the motor and neighboring region. *Ca*Kip3 residues present in this area are labeled. The figure was made with GNU R[5] and USCF Chimera[6].

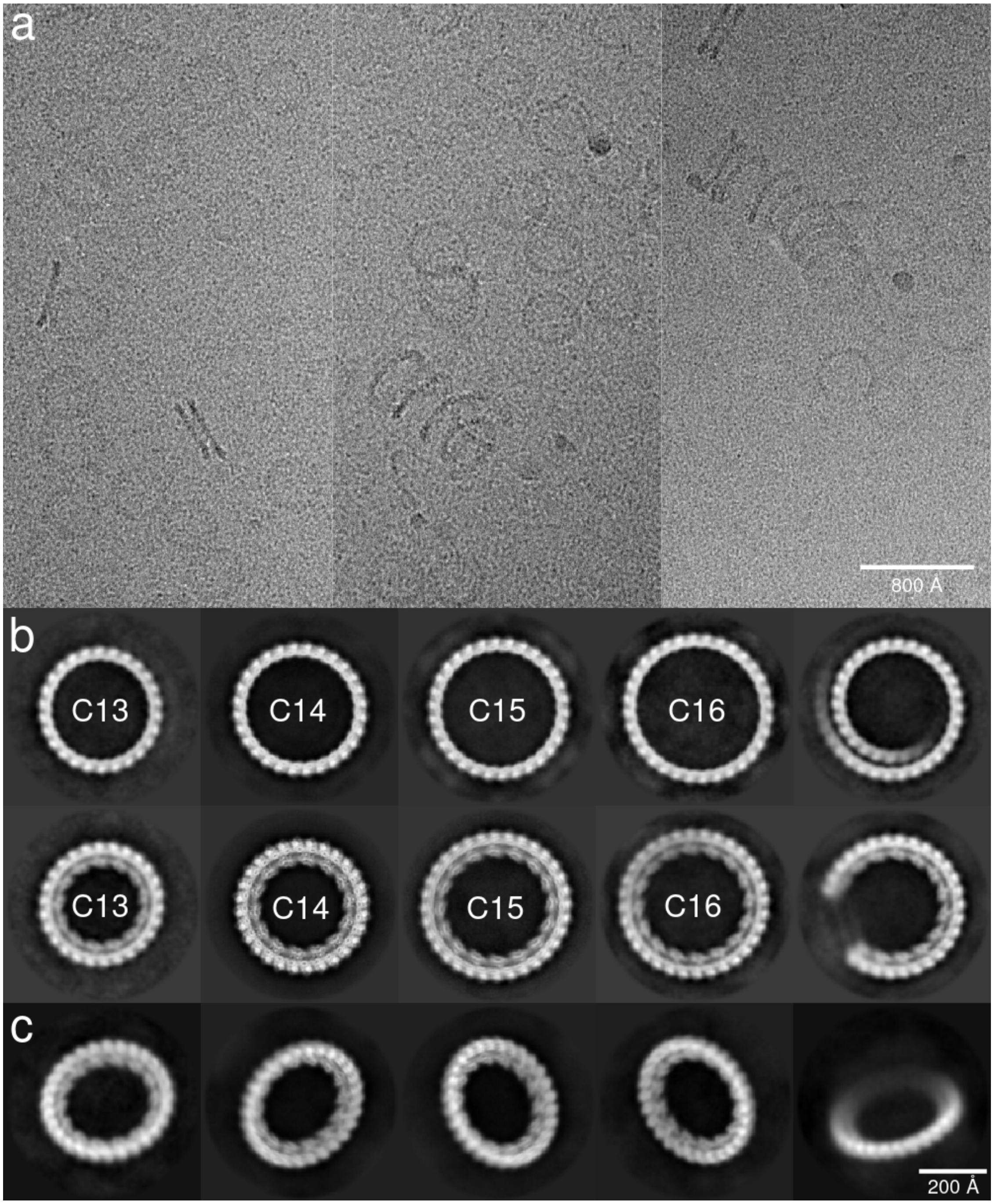

Supplementary Fig. 4: Polymer structures detected in the dolastatin-10 tubulin sample with *Ca*Kip3-MDC and 4 mM AMP-PNP. (a) Three micrograph subsets extracted from 3 distinct micrographs of the tilted CT-*Ca*Kip3-MDC-ANP dataset. These micrographs represent well the different curved tubulin polymer structures seen. Both rings and spiral/spring flexible structures are present. These types of structures were observed in all independent experiments performed on *Ca*Kip3-MDC with AMP-PNP by either cryo-EM (n=2) or negative staining EM (n=4). **(b)** Representative class averages of the different structures that were detected by 2D classification performed on the dolastatin-10 tubulin sample mixed with *Ca*Kip3-MDC and 4 mM AMP-PNP and collected with no stage tilt. This 2D classification was done after picking ring-like structures i.e., the numerous spring/spiral were avoided. These structures having strong preferential orientation in the plane of the microscope stage, the resulting 2D class averages facilitates the identification of the different structures present. The classes averages correspond to tubulin protofilament structures either undecorated (top row) or decorated with *Ca*Kip3-MDC (bottom row). This dichotomy is possibly related to a high cooperative binding behavior of *Ca*Kip3. The tubulin ring composition varies from 13 to 16 tubulin dimers; C*n* labels indicate rings with *n* tubulin dimers. The decorated C14 class is preponderant (note the more detailed class average as a consequence). The scale is the same as in panel **(c)**. **(c)** Subset of the class averages of decorated C14 rings obtained after the third cycle of autopicking/centering/2D classification done in the processing of the CT-*Ca*Kip3-MDC-ANP dataset collected with a 40-degree stage tilt. These class averages show various degrees of out of plane tilt.
The figure was made with GNU R[5].

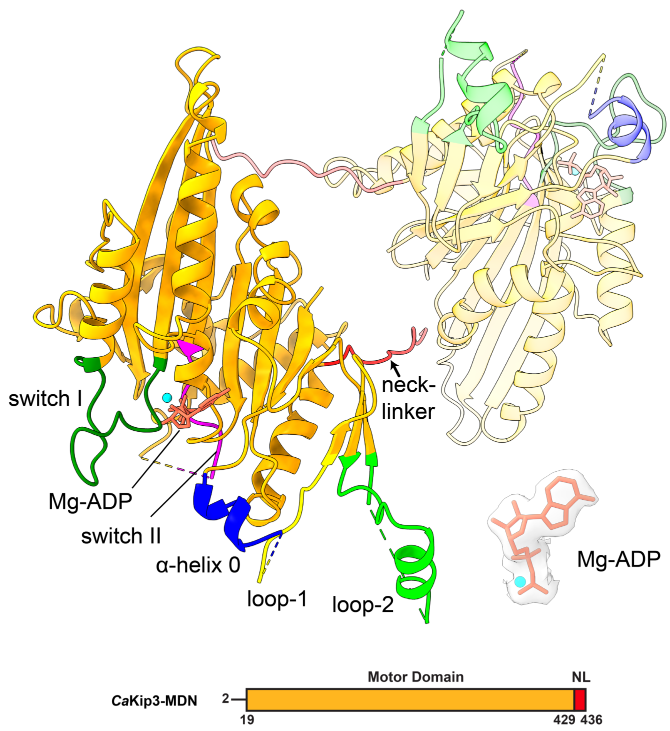

Supplementary Fig. 5: X-ray crystal structure of *Ca*Kip3-MDN. The motor core of each of the two molecules in the asymmetric unit is colored orange, one of which is shown as a transparent model. Specific regions of the motor domain that are involved in catalytic activity are colored according to **Fig. 1**. The inset panel shows the electron density for Mg-ADP from a 2mF_o − DF_c map contoured at 1.0 σ using PyMOL[7].

Structure	Distance from switch I to P-loop (192-302) (Å)	Distance from switch I to α-helix 0 (34-304) (Å)	Nucleotide-binding pocket conformation
*Ca*Kip3-MDN-ADP	12.7	23.3	semi-open
MT-*Ca*Kip3-MDC-APO	14.9	25.6	open
MT-*Ca*Kip3-MDC-ANP	14.8	25.5	open
MT-*Ca*Kip3-MDN-ANP	15.0	26.0	open
MT-*Ca*Kip3-MDC-AAF	11.3	23.5	closed
CT-*Ca*Kip3-MDC-ANP	10.6	23.7	closed
MT-*Ca*Kip3-MDN$_{\text{L2-}\textit{Hs}\text{KHC}}$-APO	14.0	25.3	open
MT-*Ca*Kip3-MDN$_{\text{L2-}\textit{Hs}\text{KHC}}$-ANP	11.1	24.1	closed

Supplementary Fig. 6: Nucleotide binding-pocket measurements for *Ca*Kip3 structures. Measurements displayed are associated with the opening and closing of the nucleotide-binding pocket[8]. Measurements were taken between the α-carbon atoms of the specified residues. Based on these distances, *Ca*Kip3 structures were categorized into three distinct groups: open (orange), closed (blue), and semi-open (green).

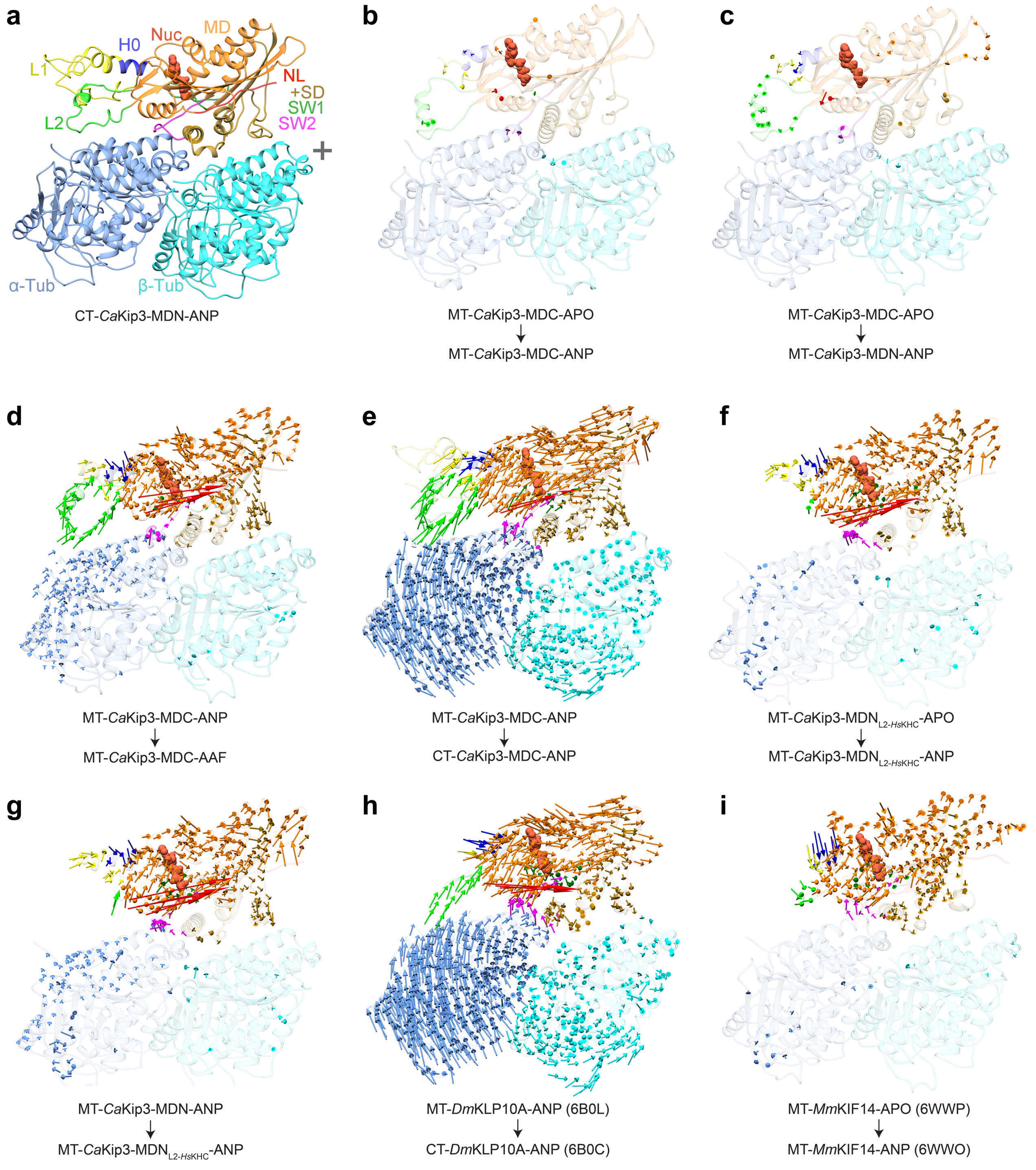

Supplementary Fig. 7: Comparison of conformational changes in kinesins and tubulin. Displacement vectors for Cα atoms in **(a-g)** *Ca*Kip3 and tubulin, **(h)** *Dm*KLP10A (kinesin-13) and tubulin, **(i)** and *Mm*KIF14 (kinesin-3) and tubulin when comparing the indicated structures. All structure comparisons were done by alignment to the β-tubulin chain. Displacement vectors for Cα atoms are colored regionally to match the segment of the protein model that is compared, using the color scheme in **Fig. 1**. Only vectors with a magnitude of 1.0 Å or higher are shown.

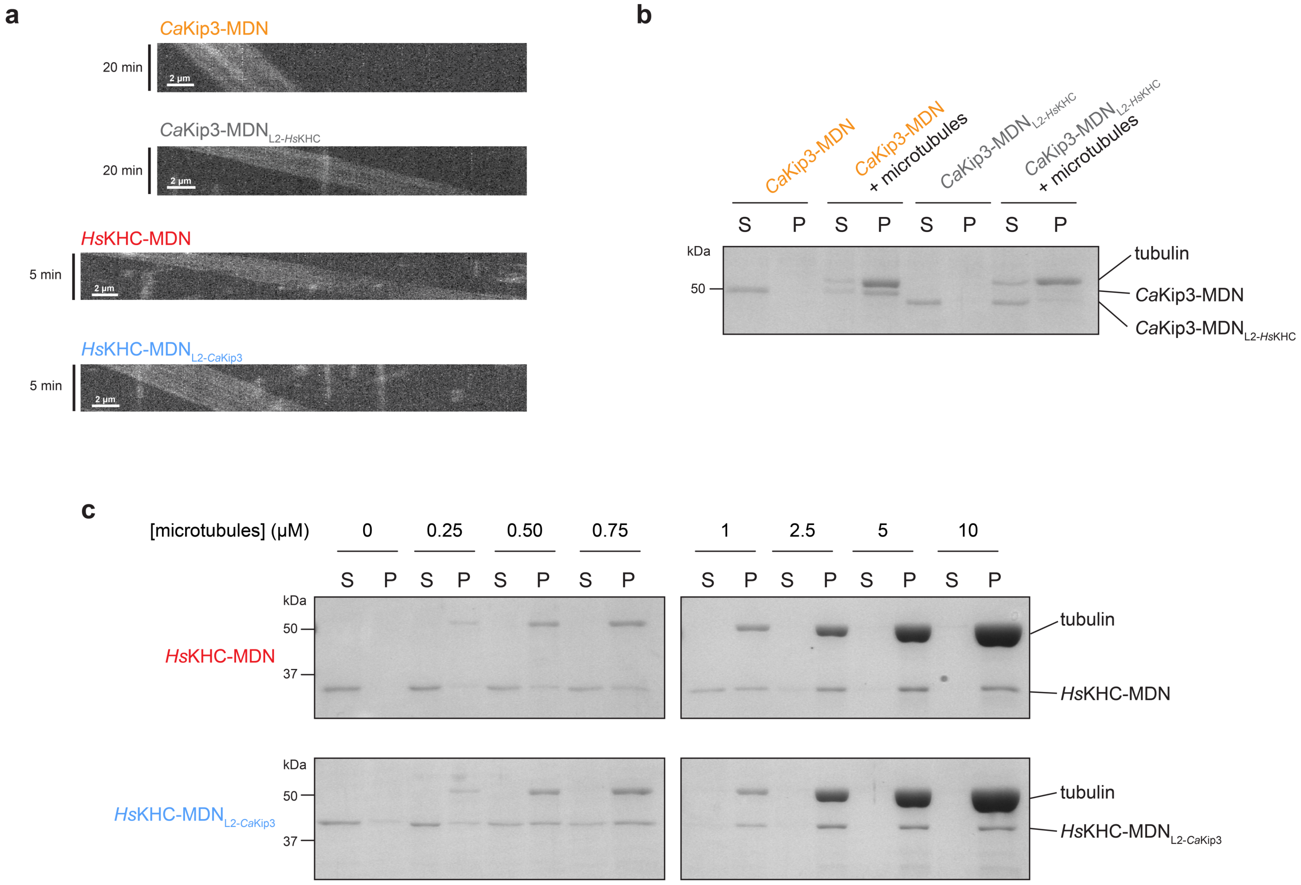

Supplementary Fig. 8: Representative data for microtubule gliding and co-sedimentation assays. (a) Representative kymographs from the microtubule gliding assay for *Ca*Kip3-MDN, *Ca*Kip3-MDN$_{L2\text{-}HsKHC}$, *Hs*KHC-MDN, and *Hs*KHC-MDN$_{L2\text{-}CaKip3}$ constructs. Frames were collected every 20 seconds over a 20-minute period for kinesin-8 constructs and every 5 seconds over a 5-minute period for kinesin-1 constructs. **(b)** Representative SDS-PAGE gels showing results of the microtubule co-sedimentation assay for *Ca*Kip3-MDN and *Ca*Kip3-MDN$_{L2\text{-}HsKHC}$. Reactions contained 1 µM kinesin, 1 µM taxol-stabilized microtubules, and 2 mM AMP-PNP. Microtubules were pelleted by centrifugation to separate the free kinesin (S) and microtubule-bound kinesin (P). SDS-PAGE followed by Coomassie brilliant blue staining was used to determine the fraction of microtubule-bound kinesin. Similar results were obtained from three independent experiments. **(c)** Microtubule co-sedimentation assay results for *Hs*KHC-MDN and *Hs*KHC-MDN$_{L2\text{-}CaKip3}$. Reactions contained 1 µM kinesin, 0-10 µM taxol-stabilized microtubules, and 2 mM AMP-PNP. Microtubules were pelleted by centrifugation to separate the free kinesin (S) and microtubule-bound kinesin (P). SDS-PAGE and Coomassie brilliant blue staining were used to determine the fraction of microtubule-bound kinesin. Samples derive from the same experiment and gels were processed in parallel. Similar results were obtained from three independent experiments. Source data are provided as a Source Data file.

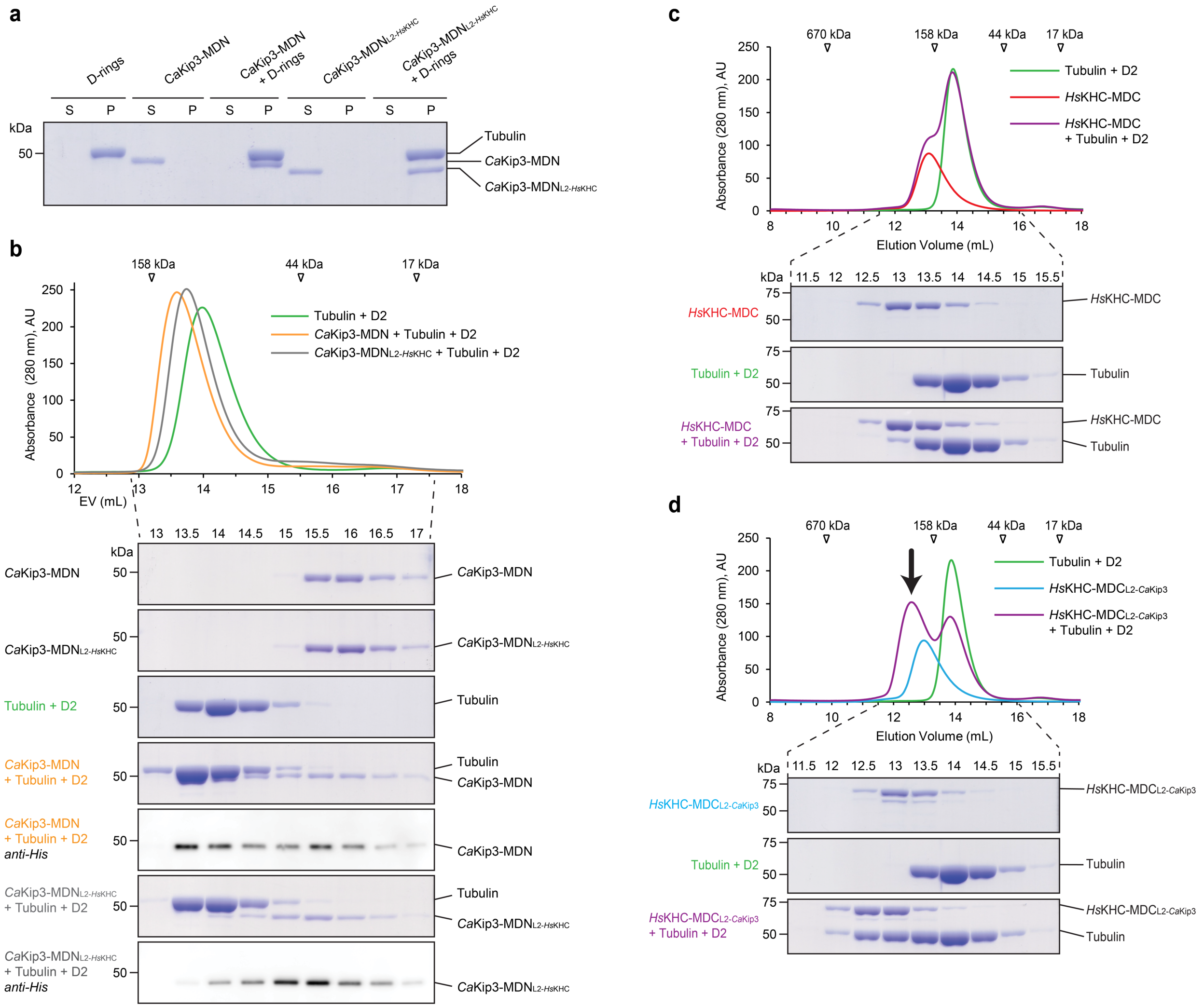

Supplementary Fig. 9: Effects of the kinesin-8 loop-2 on curved tubulin binding. (a) D-ring co-sedimentation binding assay performed on *Ca*Kip3-MDN and *Ca*Kip3-MDN$_{L2\text{-}HsKHC}$ at saturating conditions (4 µM kinesin, 4 µM D-rings, 20 µM dolastatin-10, 2 mM AMP-PNP). Reactions were incubated for 10 minutes, then subjected to ultracentrifugation. Supernatant (S) and pellet (P) fractions were analyzed via SDS-PAGE followed by Coomassie-blue-staining. Similar results were obtained from two independent experiments. **(b)** Size-exclusion chromatography (SEC) profiles for *Ca*Kip3-MDN and *Ca*Kip3-MDN$_{L2\text{-}HsKHC}$ in the presence of tubulin-DARPin-D2 (D2) and tubulin-D2 alone. Proteins were in a 1:1:1 molar ratio. Samples were supplemented with 0.2 mM AMP-PNP and applied to a Superdex 200 10/300 GL column in HEPES buffer. Chromatograms of *Ca*Kip3-MDN and *Ca*Kip3-MDN$_{L2\text{-}HsKHC}$ standards are omitted for clarity (AU = arbitrary units). SEC fractions were subjected to SDS-PAGE and stained with Coomassie blue. SEC fractions where *Ca*Kip3 could not be resolved from tubulin were additionally subjected to Western blotting analysis using anti-His antibody. Similar results were obtained from two independent experiments. **(c-d)** SEC profiles for *Hs*KHC-MDC and *Hs*KHC-MDC$_{L2\text{-}CaKip3}$ in the presence of tubulin-D2. Black arrow on (**d**) points to the chromatogram peak observed in the *Hs*KHC-MDC$_{L2\text{-}CaKip3}$ + tubulin + D2 sample that is not observed in the *Hs*KHC-MDC + tubulin + D2 sample. Sample preparation and column conditions same as in **(b)**. Similar results were obtained from two independent experiments. Source data are provided in the Source Data file.

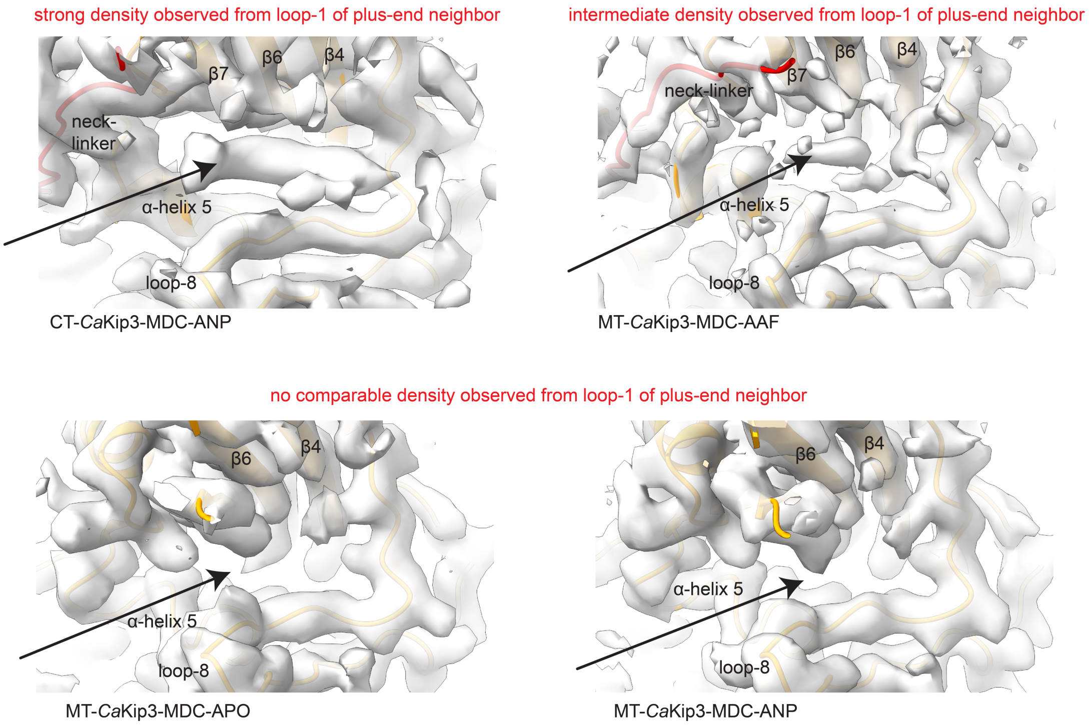

Supplementary Fig. 10: Loop-1 densities from plus-end neighboring kinesins. Densities resulting from loop-1 of the plus-end neighboring kinesin can observed strongly in the CT-*Ca*Kip3-MDC-ANP and at an intermediate intensity in the MT-*Ca*Kip3-MDC-AAF cryo-EM maps. Black arrows point to location where loop-1 densities are, or are not, observed in between the loop-8 lobe and the underside of the central β-sheet. Cryo-EM maps represented as a transparent grey surface.

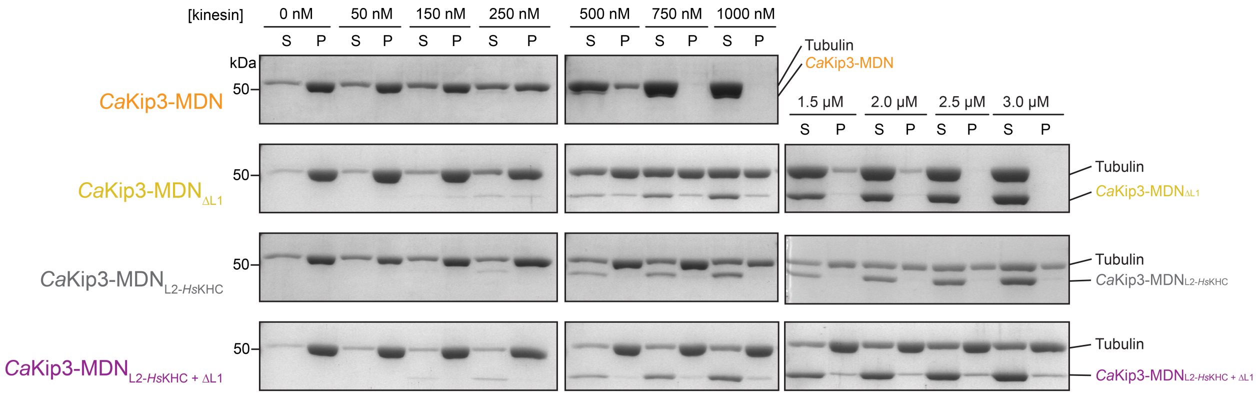

Supplementary Fig. 11: Representative data for microtubule depolymerization by sedimentation assay. Representative results are shown for *Ca*Kip3-MDN, CaKip3-MDN$_{\Delta L1}$, *Ca*Kip3-MDN$_{L2\text{-}\mathit{Hs}KHC}$, and *Ca*Kip3-MDN$_{L2\text{-}\mathit{Hs}KHC+\Delta L1}$. Reactions were performed with 2 μM GMP-CPP-stabilized microtubules, 20 mM MgATP, and 0-3 μM kinesin in BRB80 buffer. Following a 20-minute incubation, free tubulin was separated from microtubules via ultracentrifugation. Supernatant (S) and pellet (P) fractions were subject to SDS-PAGE analysis. Samples derive from the same experiment and gels were processed in parallel. Similar results were obtained from three independent experiments. Source data are provided in the Source Data file.

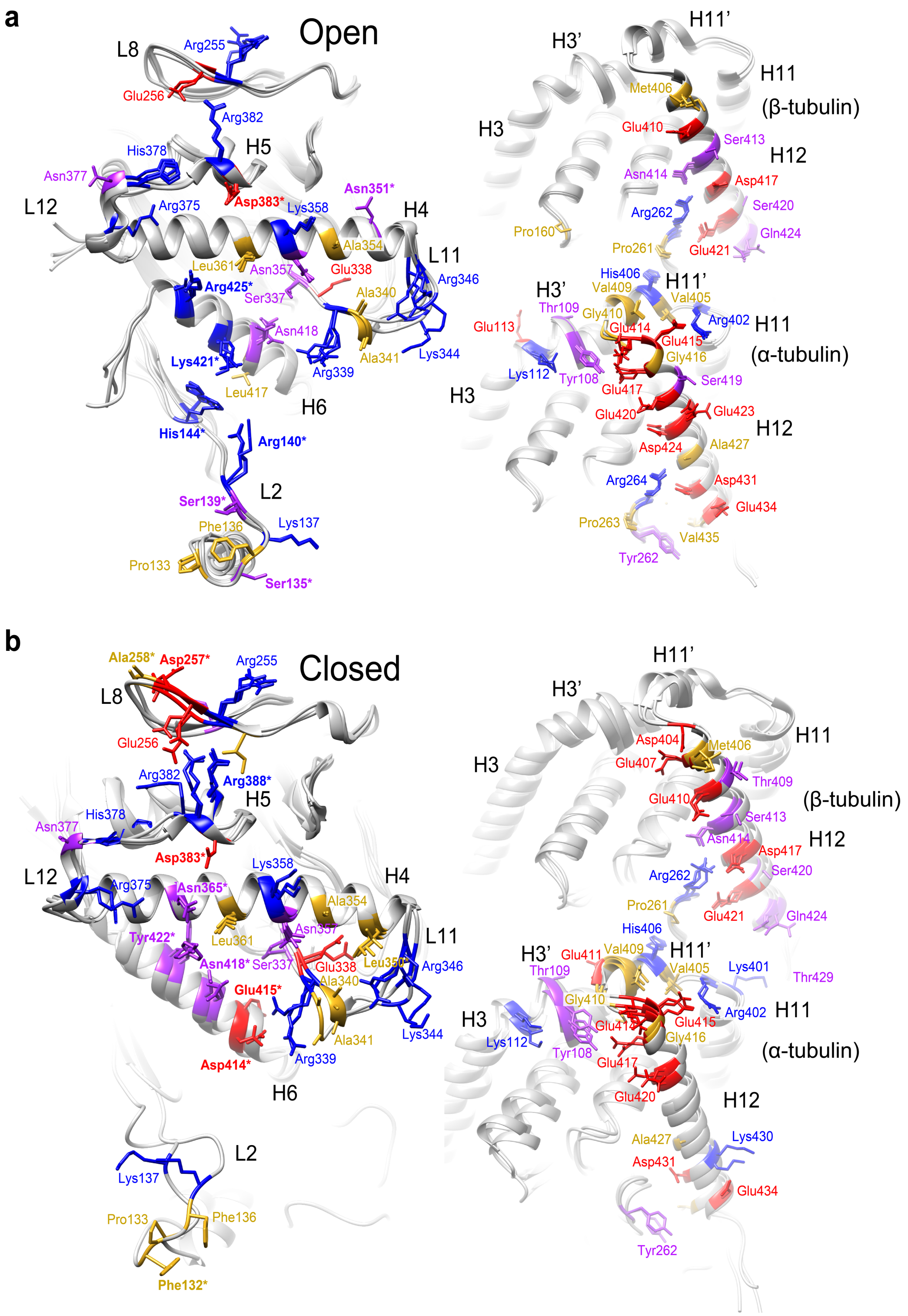

Supplementary Fig. 12: *Ca*Kip3-tubulin interacting residues. *Ca*Kip3 and tubulin residue side chains identified as making contacts by the UCSF-Chimera routine find clashes/contacts[6]. All the models exhibiting the open **(a)** or closed **(b)** conformation are superimposed. Residue side chains are colored by type (polar - purple, hydrophobic - yellow, negatively charged - red and positively charged - blue). *Ca*Kip3 residues that make contacts with tubulin in only the closed or open state are shown in bold and with an asterisk.

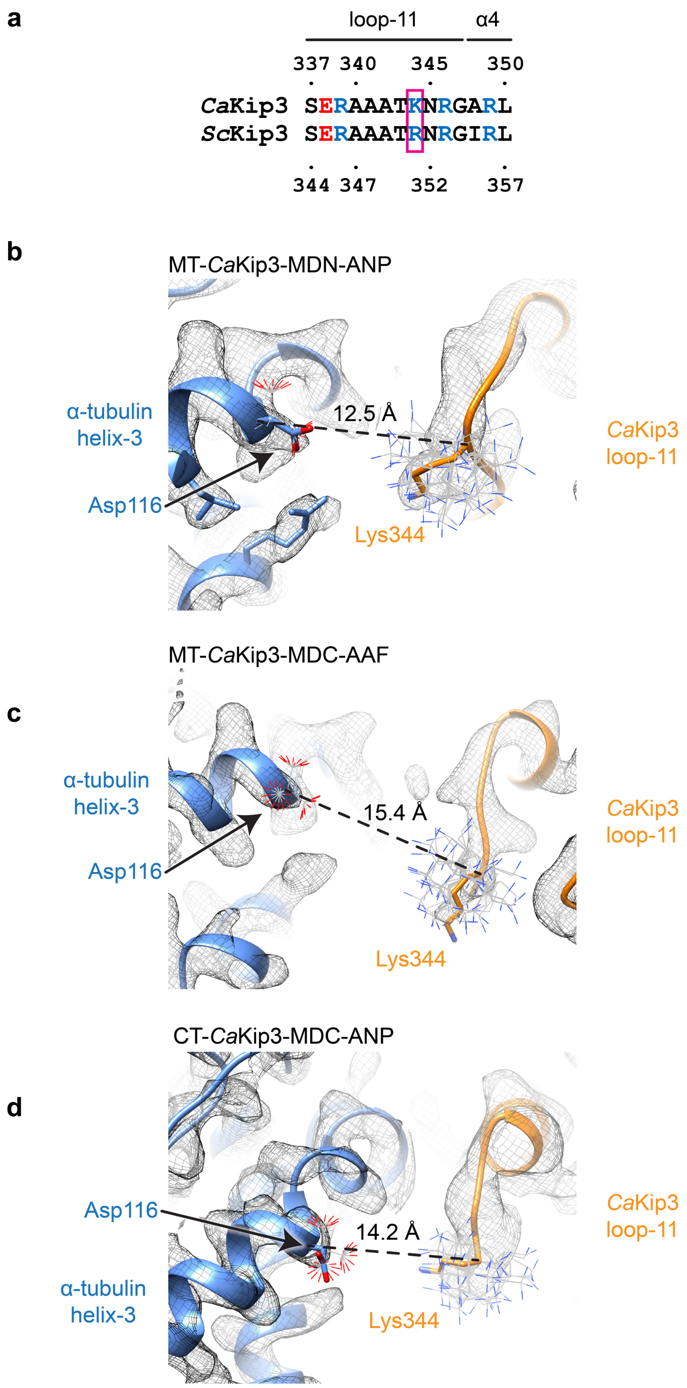

Supplementary Fig. 13: Loop-11 densities in relation to α-tubulin Asp116. (a) Sequence alignment of the loop-11 of *Ca*Kip3 and *Sc*Kip3. A magenta box encloses the candidate residue proposed to interact with Asp116 on α-tubulin (*Ca*Kip3 Lys344/*Sc*Kip3 Arg351)[9,10]. Close-up view of *Ca*Kip3's loop-11 in the **(b)** MT-*Ca*Kip3-MDN-ANP, **(c)** MT-*Ca*Kip3-MDC-AAF, and **(d)** CT-*Ca*Kip3-MDC-ANP structures. The modelled conformations of *Ca*Kip3 Lys344 and α-tubulin Asp116 are shown as sticks. All possible rotamer conformations are displayed as thinner lines to depict that no side chain conformations are within bonding distance of each other. Distances displayed are from α-carbon to α-carbon. Note that on curved tubulin, the distance between Lys344 and Asp116 increases. Cryo-EM densities are displayed as a mesh surface.

Supplementary References

1. Sherwood, R.K. & Bennett, R.J. Microtubule motor protein Kar3 is required for normal mitotic division and morphogenesis in Candida albicans. *Eukaryot Cell* **7**, 1460-74 (2008).
2. Shoukat, I., Frazer, C. & Allingham, J.S. Kinesin-5 Is Dispensable for Bipolar Spindle Formation and Elongation in Candida albicans, but Simultaneous Loss of Kinesin-14 Activity Is Lethal. *mSphere* **4**(2019).
3. Madeira, F. et al. The EMBL-EBI search and sequence analysis tools APIs in 2019. *Nucleic Acids Res* **47**, W636-W641 (2019).
4. Gouet, P., Courcelle, E., Stuart, D.I. & Metoz, F. ESPript: analysis of multiple sequence alignments in PostScript. *Bioinformatics* **15**, 305-8 (1999).
5. Ihaka, R. & Gentleman, R. R: A Language for Data Analysis and Graphics. *Journal of Computational and Graphical Statistics* **5**, 299-314 (1996).
6. Pettersen, E.F. et al. UCSF Chimera--a visualization system for exploratory research and analysis. *J Comput Chem* **25**, 1605-12 (2004).
7. Schrodinger, LLC. The PyMOL Molecular Graphics System, Version 1.3r1. (2010).
8. Benoit, M. et al. Structural basis of mechano-chemical coupling by the mitotic kinesin KIF14. *Nat Commun* **12**, 3637 (2021).
9. Arellano-Santoyo, H. et al. A Tubulin Binding Switch Underlies Kip3/Kinesin-8 Depolymerase Activity. *Dev Cell* **42**, 37-51 e8 (2017).
10. Arellano-Santoyo, H. et al. Multimodal tubulin binding by the yeast kinesin-8, Kip3, underlies its motility and depolymerization. *bioRxiv* (2021).
