# Supplementary material for: Kinesin-8-specific loop-2 controls the dual activities of the motor domain according to tubulin protofilament shape
Source: Nat Commun. 2022 Jul 20;13:4198. doi: 10.1038/s41467-022-31794-3 (PMC9300613; doi:10.1038/s41467-022-31794-3)
Supplement: Supplementary file 3 — Description of additional Supplementary File [file 41467_2022_31794_MOESM3_ESM.pdf]

## **Descriptions of Additional Supplementary Data Files**

Supplementary Movie 1: Conformational changes in CaKip3 and tubulin.
