# Supplementary material for: Kinesin-8-specific loop-2 controls the dual activities of the motor domain according to tubulin protofilament shape
Source: Nat Commun. 2022 Jul 20;13:4198. doi: 10.1038/s41467-022-31794-3 (PMC9300613; doi:10.1038/s41467-022-31794-3)
Supplement: Supplementary file 5 — Reporting Summary [file 41467_2022_31794_MOESM5_ESM.pdf]

nature portfolio

Corresponding author(s): John Allingham, Hernando Sosa

Last updated by author(s): Jun 23, 2022

Reporting Summary

Nature Portfolio wishes to improve the reproducibility of the work that we publish. This form provides structure for consistency and transparency in reporting. For further information on Nature Portfolio policies, see our Editorial Policies and the Editorial Policy Checklist.

Statistics

For all statistical analyses, confirm that the following items are present in the figure legend, table legend, main text, or Methods section.

n/a	Confirmed	
☐	☒	The exact sample size (*n*) for each experimental group/condition, given as a discrete number and unit of measurement
☐	☒	A statement on whether measurements were taken from distinct samples or whether the same sample was measured repeatedly
☐	☒	The statistical test(s) used AND whether they are one- or two-sided<br>*Only common tests should be described solely by name; describe more complex techniques in the Methods section.*
☒	☐	A description of all covariates tested
☒	☐	A description of any assumptions or corrections, such as tests of normality and adjustment for multiple comparisons
☐	☒	A full description of the statistical parameters including central tendency (e.g. means) or other basic estimates (e.g. regression coefficient) AND variation (e.g. standard deviation) or associated estimates of uncertainty (e.g. confidence intervals)
☐	☒	For null hypothesis testing, the test statistic (e.g. *F*, *t*, *r*) with confidence intervals, effect sizes, degrees of freedom and *P* value noted<br>*Give P values as exact values whenever suitable.*
☒	☐	For Bayesian analysis, information on the choice of priors and Markov chain Monte Carlo settings
☒	☐	For hierarchical and complex designs, identification of the appropriate level for tests and full reporting of outcomes
☒	☐	Estimates of effect sizes (e.g. Cohen's *d*, Pearson's *r*), indicating how they were calculated

Our web collection on statistics for biologists contains articles on many of the points above.

Software and code

Policy information about availability of computer code

Data collection	Microtubule gliding images were collected with the Olympus cellSens software (version 1.17). Western blots were imaged using an Azure C300 Digital Imager. X-ray diffraction data for the CaKip3-MDN-ADP crystals were collected using the synchrotron beamline CMCF 08ID-1 of the Canadian Light Source (Saskatoon, Canada). Cryo-EM data were collected on Titan Krios microscopes with Leginon.
Data analysis	Rates of ATP turnover by kinesins were determined using Microsoft Excel and GraphPad Prism 8.0 (GraphPad Software, San Diego, CA). Kd values for microtubule binding by kinesins was determined using GraphPad Prism 8.0. Microtubule gliding velocity by kinesins was calculated using ImageJ (NIH) and Microsoft Excel. Gaussian curves from histograms of microtubule gliding data were fit in GraphPad Prism 8.0. EC50 values for microtubule depolymerization by kinesins was determined using GraphPad Prism 8.0. For X-ray diffraction data, the in-house software program AutoProcess developed at the synchrotron beamline CMCF was used to run XDS, which processed and scaled the diffraction data and produced the reflection output file for structure determination. The initial CaKip3-MDN-ADP structure was solved by molecular replacement using MOLREP. The final model was produced after several cycles of manual building in COOT and structure refinement using PHENIX. X-ray structure model was displayed with UCSF-Chimera, Chimera X, and PyMol. Cryo-EM data were processed, analyzed, or displayed using the following software as described in the Methods or in the Figure corresponding legends: mag_distortion_estimate v1.0, MotionCor 2, Gctf, spider, eman 1, eman 2, frealign 9.11, cistem 1.0.0, relion 3.1, bsoft, localdeblur in, xmipp, modeller, rosetta, phenix, coot, Isolde, UCSF-Chimera, Chimera X, R, Python.

For manuscripts utilizing custom algorithms or software that are central to the research but not yet described in published literature, software must be made available to editors and reviewers. We strongly encourage code deposition in a community repository (e.g. GitHub). See the Nature Portfolio guidelines for submitting code & software for further information.

Data

Policy information about availability of data

All manuscripts must include a data availability statement. This statement should provide the following information, where applicable:

- Accession codes, unique identifiers, or web links for publicly available datasets
- A description of any restrictions on data availability
- For clinical datasets or third party data, please ensure that the statement adheres to our policy

Atomic coordinates of the CaKip3-MDN-ADP X-ray crystal structure have been deposited in the Protein Data Bank (PDB) under accession code 7LFF [https://www.rcsb.org/structure/7LFF] (Table 1). Atomic coordinates and corresponding cryo-EM density maps, including the half maps, masks and FSC curves used to estimate spatial resolution have been deposit-ed in the Protein Data Bank (PDB) and Electron Microscopy Data Bank (EMDB) under the accession codes 7TQX [https://www.rcsb.org/structure/7TQX], 7TQY [https://www.rcsb.org/structure/7TQY], 7TQZ [https://www.rcsb.org/structure/7TQZ], 7TR0 [https://www.rcsb.org/structure/7TR0], 7TR1 [https://www.rcsb.org/structure/7TR1], 7TR2 [https://www.rcsb.org/structure/7TR2], 7TR3 [https://www.rcsb.org/structure/7TR3] and EMD-26074 [https://www.ebi.ac.uk/emdb/EMD-26074], EMD-26075 [https://www.ebi.ac.uk/emdb/EMD-26075], EMD-26076 [https://www.ebi.ac.uk/emdb/EMD-26076], EMD-26077 [https://www.ebi.ac.uk/emdb/EMD-26077], EMD-26078 [https://www.ebi.ac.uk/emdb/EMD-26078], EMD-26079 [https://www.ebi.ac.uk/emdb/EMD-26079], EMD-26080 [https://www.ebi.ac.uk/emdb/EMD-26080]
(Table 2). Source data are provided with this paper.

Field-specific reporting

Please select the one below that is the best fit for your research. If you are not sure, read the appropriate sections before making your selection.

☒ Life sciences ☐ Behavioural & social sciences ☐ Ecological, evolutionary & environmental sciences

For a reference copy of the document with all sections, see nature.com/documents/nr-reporting-summary-flat.pdf

Life sciences study design

All studies must disclose on these points even when the disclosure is negative.

Sample size	All measurements for microtubule gliding activity involve three independent experiments per kinesin analyzed. Dozens of individual microtubules, from different microscopic fields of view, were tracked for each timelapse of microtubule gliding by each test protein. At least 100 microtubules in total were tracked for each protein tested. This sample size ensured that over 75% of all microtubules in the visible fields of view were tracked, thus representing gliding speeds of the overall population of microtubules in each condition. X-ray diffraction data analysis involved collection of hundreds of unique diffraction images from a single protein crystal, giving reflection position and intensity data for tens of thousands of reflections. To produce 3D Cryo-EM maps at the highest possible resolution (near atomic) thousands of images were collected for each experimental condition as indicated in Table 2.
Data exclusions	X-ray diffraction reflections were selected in a non-biased manner by image analysis software (XDS) and the number of reflections used for structure refinement are indicated in Table 1. Cryo-EM images were selected in a non-biased manner by image analysis software using well defined criteria (quality, microtubule type etc.). The number of particles identified as the selected microtubule type (15R) and the ones included in the final 3D reconstructions are indicated in Table 2.
Replication	All measurements for ATPase activity, microtubule binding, microtubule gliding, and microtubule depolymerization activity were done in triplicate (three independent experiments per protein prep). All attempts at replication of these experiments were successful.
Randomization	Randomization was not relevant to this study as it involved bulk analyses of biochemical reactions by purified protein molecules, which cannot be individually selected and assessed for function or structure.
Blinding	This study as it involves bulk analyses of biochemical reactions by purified proteins and structural analyses of purified proteins and protein complexes. Blinding is not applicable because no grouping was needed for the biochemical studies and blinding is not feasible for X-ray crystallography or cryoEM because visual inspection by the experimenter is necessary to ascertain data quality.

Reporting for specific materials, systems and methods

We require information from authors about some types of materials, experimental systems and methods used in many studies. Here, indicate whether each material, system or method listed is relevant to your study. If you are not sure if a list item applies to your research, read the appropriate section before selecting a response.

Materials & experimental systems

n/a	Involved in the study	
☐	☒	Antibodies
☒	☐	Eukaryotic cell lines
☒	☐	Palaeontology and archaeology
☒	☐	Animals and other organisms
☒	☐	Human research participants
☒	☐	Clinical data
☒	☐	Dual use research of concern

Methods

n/a	Involved in the study	
☒	☐	ChIP-seq
☒	☐	Flow cytometry
☒	☐	MRI-based neuroimaging

Antibodies

Antibodies used

Unlabeled anti-His antibody used for microtubule gliding assays was purchased from Thermo Fisher (cat. no. 05-949-MI, Clone: HIS.H8). HRP-conjugated anti-His antibody used for western blotting was purchased from Abcam (cat. no. ab1187).

Validation

The unlabeled Anti-Histidine Tagged Antibody from Thermo Fisher (cat. no. 05-949-MI), clone HIS.H8, is a mouse monoclonal antibody validated for use in immunocytochemistry, immunoprecipitation, western blotting for the detection of Histidine Tagged proteins. 2 μg/mL of a previous lot of antibody showed positive immunostaining for His tagged Akt1/PKBα in transfected HeLa cells. This antibody has been reported by an independent laboratory to immunoprecipitate His Tagged proteins. This antibody has been referenced in numerous publications. (e.g. Chong-Long Chua et al.PloS one, 12(2), e0171989-e0171989 (2017-02-10)).

The HRP-labeled Anti-Histidine Tagged Antibody from Abcam (cat. no. ab1187) is a rabbit polyclonal antibody validated for use in ELISA and western blotting for the detection of Histidine Tagged proteins. HRP-labeled Anti-Histidine Tagged Antibodies were immunoaffinity purified using the peptide conjugated to a solid-phase support and conjugated to horseradish peroxidase. ab1187 has been referenced in 98 publications. (e.g. Cho G et al. Structural insights into phosphatidylethanolamine formation in bacterial membrane biogenesis. Sci Rep 11:5785 (2021)).
